# National Trends in Impella Utilization for Older Adult Patients With ST-Elevation Myocardial Infarction Complicated by Cardiogenic Shock

**DOI:** 10.1016/j.jscai.2025.104056

**Published:** 2025-10-27

**Authors:** Andres Cordova Sanchez, Jesse Kane, Tanush Gupta, Hanna R. Murphy, Alyssa H. Harris, Harold L. Dauerman

**Affiliations:** a Division of Cardiology, Department of Medicine, University of Vermont Medical Center, Burlington, Vermont; b Center for Advanced Analytics and Informatics, Vizient, Irving, Texas

**Keywords:** cardiogenic shock, Impella, microaxial flow pump, percutaneous coronary intervention

## Abstract

**Background:**

Current guidelines recommend implantation of a microaxial flow pump (mAFP) for selected patients with ST-elevation myocardial infarction (STEMI) complicated by cardiogenic shock (STEMI-CS). Although the DanGer Shock trial showed a mortality benefit with mAFP, a secondary analysis suggested that older adult patients may not experience the same benefit. This study evaluated the temporal trends in mAFP utilization and hospital mortality in older (≥75 years) vs younger STEMI-CS patients.

**Methods:**

Using the International Classification of Diseases, 10th Revision (ICD-10-CM) procedure codes from 344 hospitals with continuous data between 2016 and 2024 from the Vizient Clinical Data Base, we identified 20,692 patients undergoing percutaneous coronary intervention (PCI) for STEMI-CS. Temporal trends in mAFP use and mortality were stratified by older (≥75 years) vs younger age groups. Multivariable logistic regressions identified the predictors of hospital mortality.

**Results:**

Among 20,692 STEMI-CS patients undergoing PCI, 25% were ≥75 years. Older age was the strongest independent predictor of hospital mortality for STEMI-CS patients (odds ratio, 1.87; 95% CI, 1.75-2.00). Between 2016 and 2024, mAFP use for STEMI-CS increased from 9.3% to 21.5% in patients ≥75 years old and from 14% to 25.6% in patients <75 years old (*P* value for trend = .05 and .02, respectively). Mortality declined in mAFP patients <75 years old (45% in 2016 vs 38% in 2024; *P* = .003). The mortality rate for older PCI patients with STEMI-CS and mAFP utilization was consistently above 50%.

**Conclusions:**

Approximately 1 in 5 older STEMI-CS patients received mAFP, with use more than doubling since 2016. Although mortality declined significantly in mAFP-treated younger STEMI-CS PCI patients, mortality in the older group remained approximately 60% higher than that of younger patients at all time points.

## Introduction

The DanGer Shock trial demonstrated a significant reduction in mortality with the use of a microaxial flow pump (mAFP) in patients with ST-elevation myocardial infarction complicated by cardiogenic shock (STEMI-CS) at 180 days of follow-up (45.8% vs 58.5%; hazard’s ratio: 0.74, 95% CI: 0.55-0.99).^1^ These results led to the 2025 American College of Cardiology (ACC)/American Heart Association (AHA) guidelines recommending mAFP use in select patients with STEMI and CS (class IIA).^2^ However, several factors limit the trial’s generalizability and emphasize the need for patient selection. For example, only 1 in 3 patients with STEMI-CS cases in North America meet the DanGer Shock trial’s inclusion criteria.^3^ Furthermore, in a secondary analysis of the DanGer Shock cohort, Klein et al^4^ found that the mortality benefit of mAFP interacted with the age group, and the benefit was confined to younger patients (odds ratio [OR], 0.45; 95% CI, 0.28-0.73). The interaction of older age with the best treatment strategy for patients with STEMI-CS was similarly controversial in the Shock trial and warranted exploration in large multicenter cohort studies.^5^, ^6^, ^7^, ^8^, ^9^

Notably, 1 in 5 patients enrolled in the DanGer Shock trial were ≥77 years old.^1^ Whether this is an accurate representation of the patient population with STEMI-CS receiving mAFP is unknown: Underrepresentation of the older adult population is well-described in interventional cardiology trials^5^^,^^7^; inadequate older patient representation limits definitive recommendations with respect to shock management strategies, as highlighted in a recent American Heart Association Scientific Statement regarding shock therapies among older patients.^6^ Broadly inclusive, multicenter clinical registries may provide a more accurate estimate than selective clinical trials of mAFP utilization within the older STEMI-CS patient population.^8^^,^^10^

In addition to determining the true representation of older adults in a broadly inclusive STEMI-CS population, we are not aware of any studies describing temporal trends in mAFP utilization for STEMI-CS stratified by age groups. Whether mAFP use in older adults (defined as age ≥75 years for our current study) has increased, decreased, or remained stable over the past decade remains unknown. Patients >75 years represent approximately 28% to 50% of patients with acute myocardial infarction (AMI)-CS.^11^ Older age is both a potent predictor of developing shock after AMI^12^ and a strong predictor of mortality in prior AMI-CS trials and registries.^8^^,^^11^^,^^13^^,^^14^ We used a nationally representative, broadly inclusive cohort study to determine the overall utilization, temporal trends, and hospital mortality rates for mAFP use among older vs younger STEMI-CS patients.

## Materials and methods

We identified 147,792 index patient encounters undergoing PCI for STEMI among 344 continuously reporting US academic medical centers and community hospitals between January 2016 and December 2024 from the Vizient Clinical Data Base, as previously described.^15^^,^^16^ Patients were identified using the International Classification of Diseases 10th Revision (ICD-10-CM) codes. Cardiogenic shock was defined with the ICD-10 code R57.0. The complete list of diagnostic and procedural codes used in this study is provided in Supplemental Table S1. Our study identified 20,692 patients undergoing PCI for STEMI complicated by CS (STEMI-CS) between 2016 and 2024. This study utilized deidentified, retrospective data and thus was exempt from institutional board review. Data from the Vizient Clinical Data Base used by permission of Vizient, Inc. All rights reserved.

Baseline characteristics were compared between patients presenting with STEMI complicated by CS and those without shock (Supplemental Table S2). Among STEMI-CS patients, further analyses compared younger with older patients (<75 vs ≥75 years old), including baseline characteristics, therapeutic strategies, and in hospital outcomes. Multivariable logistic regressions were performed to identify independent predictors of hospital mortality (Table 2) for patients undergoing PCI for STEMI-CS, incorporating the univariate predictors (chosen at *P* < .05) of hospital mortality, as described from the clinical variables outlined in Table 1.

**Table 1:** Baseline characteristics of patients undergoing PCI for ST-elevation myocardial infarction complicated by cardiogenic shock stratified by age.

Variables	Age <75 years (n = 15,568)	Age ≥75 years (n = 5124)	*P* value
Age, y	60.5 ± 9.5	82.1 ± 5.4	<.001
Female sex	4167 (26.8%)	2371 (46.3%)	<.001
Chronic kidney disease	3070 (19.7%)	1861 (36.3%)	<.001
Diabetes mellitus	8105 (52.1%)	2470 (48.2%)	<.001
COPD	2039 (13.1%)	709 (13.8%)	.176
Peripheral vascular disease	2031 (13.1%)	865 (16.9%)	<.001
Prior stroke/TIA	1223 (7.9%)	670 (13.1%)	<.001
Prior myocardial infarction	1939 (12.5%)	694 (13.5%)	.042
Prior PCI	1843 (11.8%)	685 (13.4%)	.004
Prior CABG procedure	468 (3.0%)	293 (5.7%)	<.001
Transfer from outside hospital	3956 (25.4%)	1375 (26.8%)	.043

Data are presented as mean ± SD for continuous measures and n (%) for categorical measures. Chi-square test for categorical variables/t-test for continuous variables. Data from the Vizient Clinical Data Base used by permission of Vizient, Inc. All rights reserved.

CABG, coronary artery bypass graft; COPD, chronic obstructive pulmonary disease; PCI, percutaneous coronary intervention; STEMI, ST-elevation myocardial infarction; TIA, transient ischemic attack.

Two temporal trends analyses were performed examining practice and hospital mortality outcomes between 2016 and 2024. The first analysis identified annual use of mAFP among index patient encounters undergoing PCI for STEMI-CS (n = 20,692) stratified by age (<75 vs ≥75 years old). The second temporal trends analysis examined annual hospital mortality trends among patients undergoing PCI for STEMI-CS who received a mAFP (21.6% of the overall STEMI-CS population, n = 4470). For hospital mortality trends in this patient group (PCI for STEMI-CS receiving mAFP), the analysis was conducted for all patients and then stratified by age <75 years vs ≥75 years.

SAS version 9.4 (SAS Institute) was used to conduct all analyses, and a *P* value < .05 was considered significant. Continuous variables are presented as mean ± SD and compared using 2-sample *t* tests. Categorical variables are presented as absolute numbers and percentages and compared using χ^2^ tests. Temporal trends in mAFP utilization were analyzed by age using linear regressions with an ordinal predictor (discharge year). Multivariate logistic regression was used to identify independent predictors of hospital mortality. Candidate covariates included all baseline demographic characteristics and comorbidity variables listed in Table 1. Each variable was first assessed in univariate analysis, and those with *P* < .05 were entered into the multivariate logistic regression model (Table 2). Potential outcome variables that may influence hospital mortality (ie, acute kidney injury) were not included in the baseline clinical variable models to confine the model to those variables measurable at hospital presentation.

**Table 2:** Independent predictors of hospital mortality among patients undergoing PCI for ST-elevation myocardial infarction complicated by cardiogenic shock.

Variables	Adjusted OR (95% CI)	*P* value
Age ≥75 y	1.87 (1.75-2.00)^a^	<.001
Female sex	1.12 (1.04-1.19)	.001
Chronic kidney disease	1.27 (1.19-1.37)	<.001
Diabetes mellitus	1.23 (1.16-1.29)	<.001
Peripheral vascular disease	1.37 (1.26-1.49)	<.001
Prior stroke/TIA	1.15 (1.04-1.27)	.009
Transfer from outside hospital	1.12 (1.04-1.20)	.002

OR, odds ratio; PCI, percutaneous coronary intervention; TIA, transient ischemic attack.

Data from the Vizient Clinical Data Base used by permission of Vizient, Inc. All rights reserved.

a Strongest independent predictor of hospital mortality.

## Results

### Study population

Among 147,792 index patient encounters with STEMI undergoing PCI, 20,692 (14%) had STEMI and STEMI-CS (Central Illustration). Compared with STEMI patients without CS, those with STEMI-CS were older, were more frequently female, and had a higher burden of comorbidities, including chronic kidney disease, diabetes mellitus, chronic obstructive pulmonary disease, and prior cerebrovascular events.

**Central Illustration:**
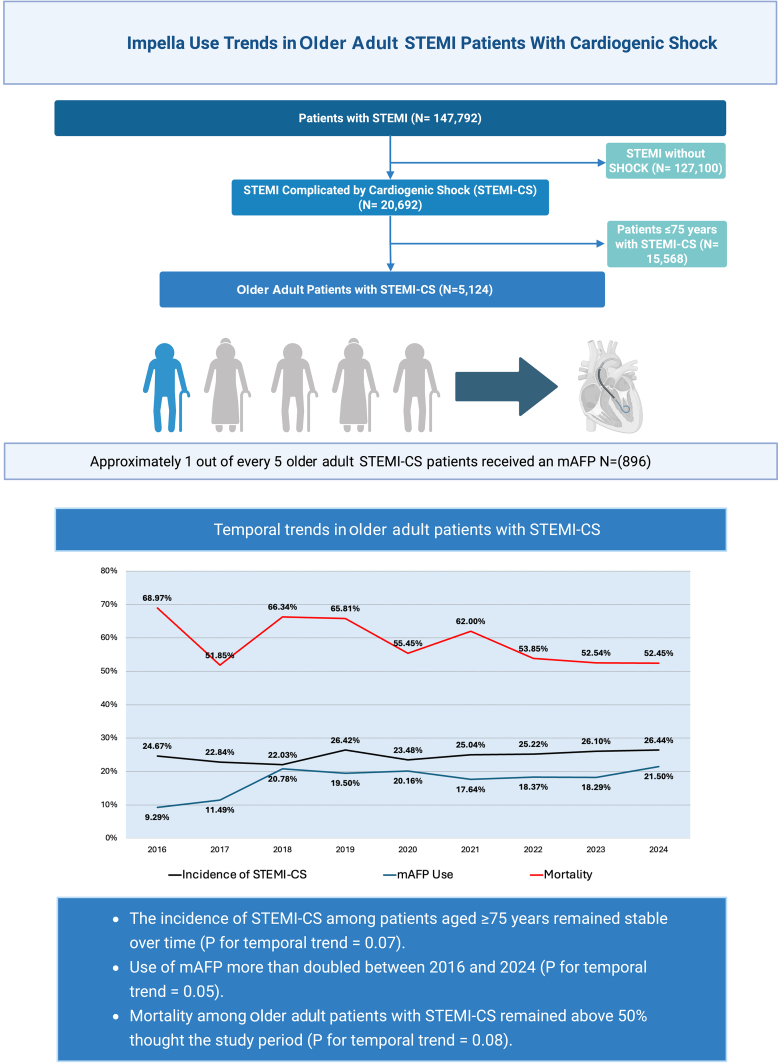
Impella use trends in a older STEMI patient with cardiogenic shock.

Patients ≥75 years comprised approximately 1 in 4 STEMI-CS PCI patients (N = 5124, 24.8%), although they comprised only 16.5% of the nonshock STEMI population (*P* < .001). The mean age of the older STEMI-CS population was 82.1 ± 5.4 years old. Peripheral vascular disease was diagnosed twice as frequently in the CS vs no CS population (14.0% vs 6.9%, *P* < .001) (Supplemental Table S2). Compared with younger STEMI-CS PCI patients, older patients were more likely to be women (46.3% vs 26.8%, *P* < .001) with increased comorbidities, including chronic kidney disease, prior stroke/transient ischemic attack, peripheral vascular disease, and prior coronary artery bypass graft (Table 1).

### Clinical management and hospital outcomes

Older STEMI-CS PCI patients were less likely to undergo invasive hemodynamic monitoring than younger STEMI-CS PCI patients (20.3% vs 24.1%, *P* < .001). Intra-aortic balloon pump (IABP) was the most commonly chosen hemodynamic support device; IABP use was similar between the younger and older groups (38.6% vs 38.9%; *P* = .67). On the other hand, mAFP was used less frequently than IABP overall; use of mAFP was less common in the older vs younger STEMI-CS populations (17.5% vs 23%; *P* < .001). ECMO was the least frequently used hemodynamic support device; use of ECMO in the older population was rare (1.5%). Management and outcomes of STEMI-CS patients are summarized in Supplemental Table S3.

Bleeding complications were frequent and occurred in approximately 30% of both younger and older STEMI-CS PCI patients (29.7% vs 27.9%, *P* = .02). Overall mortality in the entire cohort of patients with PCI for STEMI-CS was 29%. Hospital mortality was significantly higher among older vs younger STEMI-CS patients (40.1% vs 25%; *P* < .001). Univariate predictors of hospital mortality included age ≥75 years, female sex, chronic kidney disease, diabetes mellitus, peripheral vascular disease, prior stroke/transient ischemic attack, and transfer from an outside hospital. The strongest, independent predictor of hospital mortality in STEMI-CS PCI patients was age ≥ 75 years (adjusted OR, 1.87; 95% CI, 1.75-2.00) (Table 2).

### Temporal trends in mAFP utilization and hospital mortality

**Figure 1:**
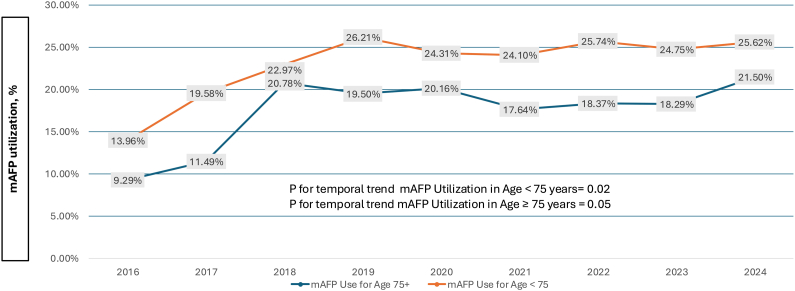
**Temporal trends in microaxial flow pump utilization among older (≥75 years) and younger (<75 years) patients undergoing percutaneous coronary intervention for STEMI complicated by cardiogenic shock.** mAFP, microaxial flow pump; STEMI, ST-elevation myocardial infarction. Data from the Vizient Clinical Data Base were used with the permission of Vizient (all rights reserved).

**Figure 2:**
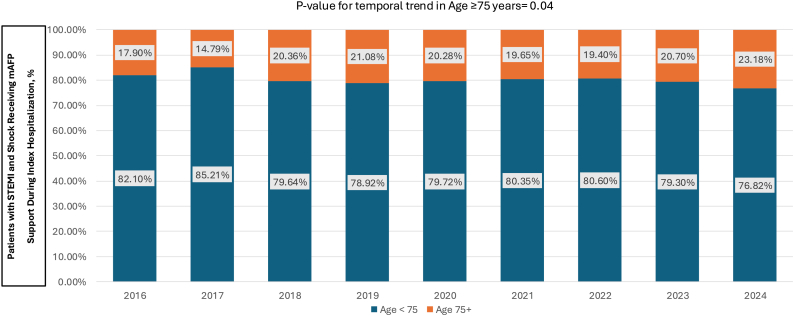
**Temporal trends in proportional microaxial flow pump utilization among all patients undergoing percutaneous coronary intervention for STEMI complicated by cardiogenic shock.** mAFP, microaxial flow pump; STEMI, ST-elevation myocardial infarction. Data from the Vizient Clinical Data Base were used with the permission of Vizient (all rights reserved).

Among the 20,692 STEMI-CS PCI patients, mAFP was utilized in 4470 (21.6%)—one-fifth of these patients were ≥75 years, and 51% were <65 years. The mAFP utilization increased over 2-fold in the older population (9.3%-21.5%, *P* for trend = .05), whereas in patients <75 years, it increased by approximately 80% (14%-25.6%, *P* for trend = .02; Figure 1). The overall proportion of primary PCI patients with CS who were older remained relatively constant (24.6%-26.4%, *P* = .07), leading to a relatively greater percentage of mAFP utilization in older patients over time (17%-23% of all mAFP patients, *P* = .04; Figure 2).

**Figure 3:**
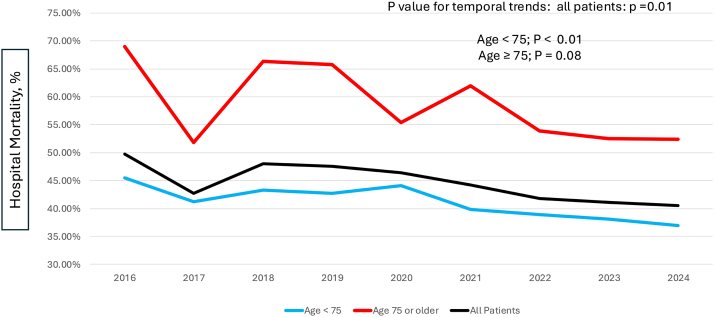
**Hospital mortality trends among percutaneous coronary intervention patients with STEMI complicated by cardiogenic shock and microaxial flow pump utilization.** STEMI, ST-elevation myocardial infarction. Data from the Vizient Clinical Data Base were used with the permission of Vizient (all rights reserved).

Hospital mortality in all STEMI-CS PCI patients treated with mAFP declined from 49.7% in 2016 to 40.5% in 2024 (*P* for temporal trend = .013; Figure 3). This reduction in hospital mortality was primarily observed among those <75 years old (45.5%-36.92%; *P* for trend = .003). Hospital mortality among older STEMI-CS patients treated with mAFP remained consistently approximately 1.5 times higher than that observed among younger patients and exceeded 50% at all time points. Temporal trends for hospital mortality among older STEMI-CS patients receiving a mAFP did not show a statistically significant reduction over the 9-year study period (*P* for trend = .08).

## Discussion

This nationwide analysis of characteristics, outcomes, and trends in STEMI-CS and mAFP utilization among 147,792 consecutive patients undergoing PCI for STEMI between 2016 and 2024 provides 3 key insights relevant to the selection of STEMI-CS patients for the potential use of mAFP: (1) patients ≥75 years present with a higher comorbidity burden and are more frequently represented in the STEMI-CS population than in the nonshock STEMI patient population. Nearly 1 in 4 STEMI-CS PCI patients are >75 years old, and thus, the interaction of older age with mAFP efficacy will be a significant clinical concern, particularly in light of increasing age-adjusted CS mortality rates since 2011^17^ despite greater mAFP use. (2) The largest relative growth in mAFP utilization over the 9-year study period was among the older STEMI-CS group. (3) Hospital mortality rates decreased from 2016 to 2024 among patients with STEMI-CS receiving mAFP, but the improvement in mortality is most apparent in the younger patient population.

### Older adult patients and STEMI-CS

We present a study population that is relevant to the older adult subgroup analysis of the DanGer Shock trial.^4^ However, our registry is more inclusive, as it is not limited by the inclusion and exclusion criteria from the clinical trial.^1^ When contrasted with the older subgroup in the DanGer Shock trial,^4^ the proportion of older STEMI-CS PCI patients was similar at approximately 25%, but the comorbidity profiles differed substantially. In our cohort, older patients had higher rates of chronic kidney disease (36% vs 14%) and diabetes mellitus (48% vs 17%) compared with the DanGer Shock trial population. Moreover, our data revealed a generally higher comorbidity burden among older compared with younger STEMI-CS patients, whereas in the DanGer Shock trial, comorbidity profiles were statistically similar across age groups. Our findings are consistent with those of prior cohort analyses of CS patients of all etiologies, which similarly demonstrate that advanced age is associated with greater illness severity, comorbidity burden, and adverse outcomes.^11^^,^^13^ The lack of interaction between comorbidity burden and older age in the DanGer Shock trial may be related to the relatively small number of older patients included (n = 81)^4^ or selection bias related to clinical trial enrollment. These differences likely reflect the selective nature of trial populations and highlight the complementary value of real-world registry data.

Older age has long been recognized as a key determinant of mortality in CS,^8^^,^^14^ and our findings reinforce this association. In our multivariate analysis of patients undergoing PCI for STEMI-CS, age ≥75 was the strongest independent predictor of hospital mortality (OR, 1.87; 95% CI, 1.75-2.00), exceeding the risk conferred by chronic kidney disease, diabetes, and peripheral vascular disease. In our cohort, older patients with STEMI-CS experienced approximately 60% higher hospital mortality compared with younger patients. The 40% hospital mortality observed in our older cohort aligns with hospital mortality rates reported in other registries.^18^

### Temporal trends in STEMI-CS mortality and mAFP utilization

The mAFP was used in 22% of all STEMI-CS cases in our cohort study. Older adults comprise only 20% of the total mAFP utilization in this study period. Temporal trends analysis demonstrates that mAFP use increased irrespective of the age group. The increasing trends in mAFP utilization for STEMI-CS observed in our study are consistent with prior registry-based analyses from the United States. In an analysis of the Medicare fee-for-service data, mAFP use increased from 9.1% to 21.5% between 2015 and 2021 (*P* < .001).^18^ Similarly, a study using the US National Inpatient Sample reported an increase in mAFP use from 4.1% in 2012 to 19.9% in 2017 (*P* < .001).^19^ These findings are consistent with earlier trends reported by Amin et al,^20^who demonstrated that mAFP use for high-risk PCI increased to nearly 32% of cases by 2016, following the device’s introduction to the market in 2008. Similarly, Almarzooq et al^18^ also reported an increase in mAFP in Medicare beneficiaries age 65-74 and age >74 years from 2015 to 2021.

We observed a marked increase in mAFP utilization for older patients between 2016 and 2024 (9% vs 22%; *P* for trend of .05). Although our study is consistent with these prior temporal trend observations, the current findings are unique in that the prior studies (1) do not include patients less than 65 years old in their younger comparison group,^18^ a cohort that comprised 51% of mAFP implantations in our findings, and (2) are not focused on the DanGer Shock trial population of STEMI-CS and thus include trends in AMI-CS or high-risk PCI Impella utilization.^19^^,^^20^ Importantly, older STEMI-CS patients experienced the largest proportional increase in mAFP use over our 9-year study period, with a 2.3-fold rise compared with a 1.8-fold increase in the younger group. This difference in growth occurred despite a stable incidence of STEMI-CS per age group and led to a 30% increase in the proportion of older patients among all mAFP-treated STEMI-CS cases. The rapidly growing use of mAFP in older STEMI-CS patients is of particular importance, given the DanGer Shock trial results^4^ and the ACC/AHA guideline recommendations for selective use of mAFP.^2^

In our study, hospital mortality among STEMI-CS patients treated with mAFP declined over time. However, this benefit was more pronounced in the younger population. Older patients receiving mAFP continued to experience substantially higher hospital mortality rates, with no statistically significant improvement over time. These results align with those presented by Klein et al,^4^ who reported that the mortality benefit derived from mAFP in the DanGer Shock trial was confined to the younger population and with those from Almarzooq et al,^18^ who observed stable mortality rates in older Medicare patients receiving mAFP between 2015 and 2021. These findings have 2 potential implications: first, older age could be an important factor in the selection of STEMI-CS patients for mAFP therapy, underscoring the need for age-specific risk stratification when considering advanced mechanical circulatory support as well as further understanding of risk categories within the older subgroup (ie, frailty indexes). Second, these findings also suggest that current mAFP technology may not adequately address the complex comorbidity burden associated with advanced age, suggesting the need for tailored technological enhancements and patient selection criteria in order to reduce mortality in this high-risk group of patients.

## Study limitations

The Vizient Clinical Database overrepresents US academic medical centers and may capture more critically ill patients than community centers, which could explain the slightly higher incidence of STEMI-CS in our cohort compared with prior studies (14% vs 10%).^21^^,^^22^ As with all administrative data sets, misclassification due to coding variability is possible. About one-quarter of patients were transferred from outside institutions, all of whom underwent PCI at the receiving hospital; transfer times and referring-hospital treatments were unavailable. Although our study focuses on the overall subgroup characteristics of the DanGer Shock analysis of older patients,^4^ the dataset cannot replicate trial-specific inclusion/exclusion criteria or long-term follow-up. We defined older adults as age ≥75 years (vs ≥77 years in DanGer Shock) to align with other shock studies.^6^ Although older age was the strongest independent predictor of hospital mortality, unmeasured confounders, including shock severity, vasopressor requirements, timing of interventions, lactate levels, intubation status, frailty, and cognitive function, may have influenced both treatment decisions and outcomes. Similarly, we could not account for potential age-related delays in medical care that may be related to frailty, dementia, do-not-resuscitate status, or the need for caregiver support in decision-making. These system-related delays could disproportionately affect outcomes in the older adult population. Finally, bleeding complications were identified using administrative ICD-10 codes, which do not distinguish between major and minor events or allow attribution to device-related complications. Thus, bleeding outcomes should be interpreted with caution.

The strength of our study population is that it is inclusive of all patients undergoing primary PCI for STEMI with CS between 2016 and 2024 at over 344 hospitals in the United States. Our findings are applicable to this registry population only, and trends may be different for patients who are ineligible for primary PCI, patients with non-ST-elevation myocardial infarction, and those with non–shock-associated MI complications.

## Conclusion

This broadly inclusive national cohort analysis highlights markedly increased utilization of mAFP among older adult patients undergoing PCI for STEMI complicated with CS. As expected, older STEMI-CS patients demonstrated a greater burden of comorbidities compared with younger patients and had significantly higher mortality than younger patients. Although mortality declined significantly in mAFP-treated younger STEMI-CS PCI patients, mortality in the older cohort remained approximately 60% higher than that of younger patients at all time points. Future studies and mAFP device iterations should prioritize age-specific risk stratification and be tailored to the unique comorbidity burden that characterizes older patients with STEMI-CS.

## Declaration of competing interest

The authors declared no potential conflicts of interest with respect to the research, authorship, and/or publication of this article.

## Supplementary material

To access the supplementary material accompanying this article, visit the online version of the *Journal of the Society for Cardiovascular Angiography & Interventions* at 10.1016/j.jscai.2025.104056.
