# Supplementary material for: National Trends in Impella Utilization for Older Adult Patients With ST-Elevation Myocardial Infarction Complicated by Cardiogenic Shock
Source: J Soc Cardiovasc Angiogr Interv. 2025 Oct 27;5(1):104056. doi: 10.1016/j.jscai.2025.104056 (PMC13033803; doi:10.1016/j.jscai.2025.104056)
Supplement: Supplementary Material [file mmc1.docx]

**Supplementary Table 1. ICD-10 Codes Used for Variable Definitions**

| **Variable** | **ICD-10-CM or ICD-10-PCS Code** |
| --- | --- |
| Bleeding | I97621, I97638, D62, L7602, L7612, L7622, L7632, M96821, M96811, M96831, M96841, 30243N0, 30243N1, 30243P0, 30243P1, 30243H0, 30243H1, 30240N0, 30240N1, 30240P0, 30240P1, 30240H0, 30240H1, 30230H0, 30230H1, 30230N0, 30230N1, 30230P0, 30230P1, 30233N0, 30233N1, 30233P0, 30233P1, K661, K2211, K250, K252, K254, K256, K2901, K2921, K2931, K2941, K2951, K2961, K2971, K2981, K2991, K260, K262, K264, K266, K270, K272, K274, K276, K5701, K5711, K5713, K5721, K5731, K5733, K5741, K5751, K5753, K5781, K5791, K5793, K51011, K51211, K51311, K51411, K51511, K51811, K51911, K50011, K50111, K50811, K50911, K625, K5521, R310, R319, R042, R58, I60, I600, I6000, I6001, I6002, I601, I6010, I6011, I6012, I602, I603, I6030, I6031, I6032, I604, I605, I6050, I6051, I6052, I606, I607, I608, I609, I61, I610, I611, I612, I613, I614, I615, I616, I618, I619, I62, I620, I6200, I6201, I6202, I6203, I621, I629, I690, I6900, I691, I6910, I692, I6920 |
| CABG | 0210083, 0210088, 0210089, 021008C, 021008F, 021008W, 0210093, 0210098, 0210099, 021009C, 021009F, 021009W, 02100A3, 02100A8, 02100A9, 02100AC, 02100AF, 02100AW, 02100J3, 02100J8, 02100J9, 02100JC, 02100JF, 02100JW, 02100K3, 02100K8, 02100K9, 02100KC, 02100KF, 02100KW, 02100Z3, 02100Z8, 02100Z9, 02100ZC, 02100ZF, 0210344, 02103D4, 0210444, 0210483, 0210488, 0210489, 021048C, 021048F, 021048W, 0210493, 0210498, 0210499, 021049C, 021049F, 021049W, 02104A3, 02104A8, 02104A9, 02104AC, 02104AF, 02104AW, 02104D4, 02104J3, 02104J8, 02104J9, 02104JC, 02104JF, 02104JW, 02104K3, 02104K8, 02104K9, 02104KC, 02104KF, 02104KW, 02104Z3, 02104Z8, 02104Z9, 02104ZC, 02104ZF, 0211083, 0211088, 0211089, 021108C, 021108F, 021108W, 0211093, 0211098, 0211099, 021109C, 021109F, 021109W, 02110A3, 02110A8, 02110A9, 02110AC, 02110AF, 02110AW, 02110J3, 02110J8, 02110J9, 02110JC, 02110JF, 02110JW, 02110K3, 02110K8, 02110K9, 02110KC, 02110KF, 02110KW, 02110Z3, 02110Z8, 02110Z9, 02110ZC, 02110ZF, 0211344, 02113D4, 0211444, 0211483, 0211488, 0211489, 021148C, 021148F, 021148W, 0211493, 0211498, 0211499, 021149C, 021149F, 021149W, 02114A3, 02114A8, 02114A9, 02114AC, 02114AF, 02114AW, 02114D4, 02114J3, 02114J8, 02114J9, 02114JC, 02114JF, 02114JW, 02114K3, 02114K8, 02114K9, 02114KC, 02114KF, 02114KW, 02114Z3, 02114Z8, 02114Z9, 02114ZC, 02114ZF, 0212083, 0212088, 0212089, 021208C, 021208F, 021208W, 0212093, 0212098, 0212099, 021209C, 021209F, 021209W, 02120A3, 02120A8, 02120A9, 02120AC, 02120AF, 02120AW, 02120J3, 02120J8, 02120J9, 02120JC, 02120JF, 02120JW, 02120K3, 02120K8, 02120K9, 02120KC, 02120KF, 02120KW, 02120Z3, 02120Z8, 02120Z9, 02120ZC, 02120ZF, 0212344, 02123D4, 0212444, 0212483, 0212488, 0212489, 021248C, 021248F, 021248W, 0212493, 0212498, 0212499, 021249C, 021249F, 021249W, 02124A3, 02124A8, 02124A9, 02124AC, 02124AF, 02124AW, 02124D4, 02124J3, 02124J8, 02124J9, 02124JC, 02124JF, 02124JW, 02124K3, 02124K8, 02124K9, 02124KC, 02124KF, 02124KW, 02124Z3, 02124Z8, 02124Z9, 02124ZC, 02124ZF, 0213083, 0213088, 0213089, 021308C, 021308F, 021308W, 0213093, 0213098, 0213099, 021309C, 021309F, 021309W, 02130A3, 02130A8, 02130A9, 02130AC, 02130AF, 02130AW, 02130J3, 02130J8, 02130J9, 02130JC, 02130JF, 02130JW, 02130K3, 02130K8, 02130K9, 02130KC, 02130KF, 02130KW, 02130Z3, 02130Z8, 02130Z9, 02130ZC, 02130ZF, 0213483, 0213488, 0213489, 021348C, 021348F, 021348W, 0213493, 0213498, 0213499, 021349C, 021349F, 021349W, 02134A3, 02134A8, 02134A9, 02134AC, 02134AF, 02134AW, 02134D4, 02134J3, 02134J8, 02134J9, 02134JC, 02134JF, 02134JW, 02134K3, 02134K8, 02134K9, 02134KC, 02134KF, 02134KW, 02134Z3, 02134Z8, 02134Z9, 02134ZC, 02134ZF |
| Cardiogenic Shock | R570 |
| Chronic Kidney Disease | Z992, Z940, Z9115, Z4932, Z4931, Z4902, Z4901, R880, N189, N186, N181, N185, N184, N183, N182, N1830, N1831, N1832 |
| Chronic Obstructive Pulmonary Disease | J44, J440, J441, J449, J439 |
| Diabetes Mellitus | E089, E099, E109, E119, E139, R7301, R7302, R7303, R7309, R739, R81, R824, Z4681, Z9641, E0800, E0801, E0810, E0811, E0821, E0822, E0829, E08311, E08319, E08319, E08321, E083211, E083211, E083212, E083213, E083213, E083219, E083219, E08329, E083291, E083291, E083292, E083292, E083293, E083293, E083299, E083299, E08331, E083311, E083312, E083312, E083313, E083313, E083319, E083319, E08339, E08339, E083391, E083392, E083393, E083399, E083399, E08341, E083411, E083412, E083412, E083413, E083413, E083419, E08349, E08349, E083491, E083491, E083492, E083492, E083493, E083499, E083499, E08351, E08351, E083511, E083511, E083512, E083512, E083513, E083519, E083521, E083522, E083523, E083529, E083531, E083532, E083532, E083533, E083539, E083539, E083541, E083541, E083542, E083542, E083543, E083549, E083549, E083551, E083552, E083553, E083559, E08359, E083591, E083592, E083593, E083599, E0836, E0836, E0837X1, E0837X1, E0837X2, E0837X2, E0837X3, E0837X9, E0837X9, E0839, E0840, E0841, E0842, E0843, E0844, E0849, E0851, E0852, E0859, E0859, E08610, E08618, E08620, E08620, E08621, E08622, E08622, E08628, E08630, E08638, E08641, E08649, E0865, E0869, E088, E0900, E0901, E0910, E0911, E0921, E0921, E0922, E0929, E0929, E09311, E09319, E09319, E09321, E093211, E093212, E093213, E093219, E09329, E09329, E093291, E093292, E093293, E093299, E09331, E093311, E093312, E093313, E093319, E09339, E093391, E093392, E093393, E093399, E09341, E093411, E093412, E093413, E093419, E09349, E093491, E093492, E093493, E093499, E09351, E093511, E093512, E093513, E093519, E093521, E093522, E093523, E093529, E093531, E093532, E093533, E093539, E093541, E093542, E093543, E093549, E093551, E093552, E093553, E093559, E09359, E093591, E093592, E093593, E093599, E0936, E0937X1, E0937X2, E0937X3, E0937X9, E0939, E0940, E0941, E0942, E0943, E0944, E0949, E0951, E0952, E0952, E0959, E09610, E09618, E09620, E09621, E09622, E09628, E09630, E09638, E09641, E09649, E0965, E0969, E098, E1010, E1011, E1021, E1022, E1029, E10311, E10319, E10321, E103211, E103212, E103213, E103219, E10329, E103291, E103292, E103293, E103299, E10331, E103311, E103312, E103313, E103319, E10339, E103391, E103391, E103392, E103393, E103399, E10341, E103411, E103412, E103413, E103419, E10349, E103491, E103492, E103493, E103499, E10351, E103511, E103512, E103513, E103519, E103521, E103522, E103523, E103529, E103531, E103532, E103533, E103539, E103541, E103542, E103543, E103549, E103551, E103552, E103553, E103559, E10359, E103591, E103592, E103593, E103599, E1036, E1037X1, E1037X2, E1037X3, E1037X9, E1039, E1040, E1041, E1041, E1042, E1043, E1044, E1049, E1051, E1052, E1059, E10610, E10618, E10620, E10621, E10622, E10628, E10630, E10638, E10638, E10641, E10649, E1065, E1069, E108, E1100, E1101, E1101, E1121, E1122, E1129, E11311, E11319, E11321, E113211, E113212, E113213, E113219, E11329, E113291, E113292, E113293, E113299, E11331, E113311, E113312, E113313, E113319, E11339, E113391, E113392, E113393, E113399, E11341, E113411, E113412, E113413, E113419, E11349, E113491, E113492, E113493, E113499, E11351, E113511, E113512, E113513, E113519, E113521, E113522, E113523, E113529, E113531, E113531, E113532, E113533, E113539, E113541, E113542, E113543, E113549, E113551, E113552, E113553, E113553, E113559, E11359, E113591, E113592, E113593, E113599, E1136, E1137X1, E1137X2, E1137X3, E1137X9, E1139, E1140, E1141, E1142, E1143, E1144, E1149, E1151, E1152, E1159, E11610, E11618, E11620, E11621, E11622, E11628, E11630, E11638, E11641, E11649, E1165, E1169, E118, E1300, E1301, E1310, E1311, E1321, E1322, E1322, E1329, E13311, E13319, E13321, E133211, E133212, E133213, E133219, E13329, E133291, E133292, E133293, E133299, E13331, E133311, E133312, E133313, E133319, E13339, E133391, E133392, E133393, E133399, E13341, E133411, E133412, E133413, E133419, E13349, E133491, E133492, E133493, E133493, E133499, E13351, E13351, E133511, E133512, E133513, E133519, E133521, E133522, E133523, E133529, E133531, E133532, E133533, E133533, E133539, E133539, E133541, E133542, E133543, E133549, E133551, E133552, E133553, E133559, E13359, E133591, E133592, E133593, E133593, E133599, E1336, E1337X1, E1337X2, E1337X3, E1337X3, E1337X9, E1339, E1340, E1341, E1342, E1343, E1344, E1349, E1351, E1352, E1359, E13610, E13618, E13620, E13621, E13622, E13628, E13630, E13638, E13641, E13649, E1365, E1369, E138 |
| Hemodialysis | Z992, 5A1D70Z, 5A1D80Z, 5A1D90Z, 5A1D00Z, 5A1D60Z |
| ECMO | 5A15223, 5A1522F, 5A1522G, 5A1522H |
| IABP | 5A02110, 5A02210 |
| mAFP | 02HA3RZ, 02HA3RS, 02HL3DZ, 5A0211D, 5A0221D |
| Implant LVAD | 02HA0QZ, 02WA0QZ |
| Left Heart Catheter | 4A020N7, 4A023N7 |
| Mechanical Ventilation | 0BH17EZ, 0BH18EZ |
| Pulmonary Artery Catheter | 02HP30Z, 02HP32Z, 02HP40Z, 02HP42Z, 02HQ30Z, 02HQ32Z, 02HQ40Z, 02HQ42Z, 02HR30Z, 02HR32Z, 02HR40Z, 02HR42Z, 4A023N6, 4A03353, 4A033B3, 4A033J3, 4A033JC, 4A043R1, 4A04XR1, 4A12X9Z, 4A13353, 4A1335C, 4A133B3, 4A133J3, 4A133JC, 4A143R0, 4A143R2, 4A143R3, 4A020N6 |
| PCI – Multi Vessel | 0271346, 027134Z, 0271356, 027135Z, 0271366, 027136Z, 0271376, 027137Z, 02713D6, 02713DZ, 02713E6, 02713EZ, 02713F6, 02713FZ, 02713G6, 02713GZ, 02713T6, 02713TZ, 02713Z6, 02713ZZ, 0271446, 027144Z, 0271456, 027145Z, 0271466, 027146Z, 0271476, 027147Z, 02714D6, 02714DZ, 02714E6, 02714EZ, 02714F6, 02714FZ, 02714G6, 02714GZ, 02714T6, 02714TZ, 02714Z6, 02714ZZ, 0272346, 027234Z, 0272356, 027235Z, 0272366, 027236Z, 0272376, 027237Z, 02723D6, 02723DZ, 02723E6, 02723EZ, 02723F6, 02723FZ, 02723G6, 02723GZ, 02723T6, 02723TZ, 02723Z6, 02723ZZ, 0272446, 027244Z, 0272456, 027245Z, 0272466, 027246Z, 0272476, 027247Z, 02724D6, 02724DZ, 02724E6, 02724EZ, 02724F6, 02724FZ, 02724G6, 02724GZ, 02724T6, 02724TZ, 02724Z6, 02724ZZ, 0273346, 027334Z, 0273356, 027335Z, 0273366, 027336Z, 0273376, 027337Z, 02733D6, 02733DZ, 02733E6, 02733EZ, 02733F6, 02733FZ, 02733G6, 02733GZ, 02733T6, 02733TZ, 02733Z6, 02733ZZ, 0273446, 027344Z, 0273456, 027345Z, 0273466, 027346Z, 0273476, 027347Z, 02734D6, 02734DZ, 02734E6, 02734EZ, 02734F6, 02734FZ, 02734G6, 02734GZ, 02734T6, 02734TZ, 02734Z6, 02734ZZ, 02C13Z6, 02C13Z7, 02C13ZZ, 02C14Z6, 02C14ZZ, 02C23Z6, 02C23Z7, 02C23ZZ, 02C24Z6, 02C24ZZ, 02C33Z6, 02C33Z7, 02C33ZZ, 02C34Z6, 02C34ZZ |
| PCI – Single Vessel | 0270346, 027034Z, 0270356, 027035Z, 0270366, 027036Z, 0270376, 027037Z, 02703D6, 02703DZ, 02703E6, 02703EZ, 02703F6, 02703FZ, 02703G6, 02703GZ, 02703T6, 02703TZ, 02703Z6, 02703ZZ, 0270446, 027044Z, 0270456, 027045Z, 0270466, 027046Z, 0270476, 027047Z, 02704D6, 02704DZ, 02704E6, 02704EZ, 02704F6, 02704FZ, 02704G6, 02704GZ, 02704T6, 02704TZ, 02704Z6, 02704ZZ, 02C03Z6, 02C03Z7, 02C03ZZ, 02C04Z6, 02C04ZZ |
| Peripheral Vascular Disease | A5200, A5201, A5202, A5209, I700, I701, I70201, I70202, I70203, I70208, I70209, I70211, I70212, I70213, I70218, I70219, I70221, I70222, I70223, I70228, I70229, I70231, I70232, I70233, I70234, I70235, I70238, I70239, I70241, I70242, I70243, I70244, I70245, I70248, I70249, I7025, I70261, I70262, I70263, I70268, I70269, I70291, I70292, I70293, I70298, I70299, I70301, I70302, I70303, I70308, I70309, I70311, I70312, I70313, I70318, I70319, I70321, I70322, I70323, I70328, I70329, I70331, I70332, I70333, I70334, I70335, I70338, I70339, I70341, I70342, I70343, I70344, I70345, I70348, I70349, I7035, I70361, I70362, I70363, I70368, I70369, I70391, I70392, I70393, I70398, I70399, I70401, I70402, I70403, I70408, I70409, I70411, I70412, I70413, I70418, I70419, I70421, I70422, I70423, I70428, I70429, I70431, I70432, I70433, I70434, I70435, I70438, I70439, I70441, I70442, I70443, I70444, I70445, I70448, I70449, I7045, I70461, I70462, I70463, I70468, I70469, I70491, I70492, I70493, I70498, I70499, I70501, I70502, I70503, I70508, I70509, I70511, I70512, I70513, I70518, I70519, I70521, I70522, I70523, I70528, I70529, I70531, I70532, I70533, I70534, I70535, I70538, I70539, I70541, I70542, I70543, I70544, I70545, I70548, I70549, I7055, I70561, I70562, I70563, I70568, I70569, I70591, I70592, I70593, I70598, I70599, I70601, I70602, I70603, I70608, I70609, I70611, I70612, I70613, I70618, I70619, I70621, I70622, I70623, I70628, I70629, I70631, I70632, I70633, I70634, I70635, I70638, I70639, I70641, I70642, I70643, I70644, I70645, I70648, I70649, I7065, I70661, I70662, I70663, I70668, I70669, I70691, I70692, I70693, I70698, I70699, I70701, I70702, I70703, I70708, I70709, I70711, I70712, I70713, I70718, I70719, I70721, I70722, I70723, I70728, I70729, I70731, I70732, I70733, I70734, I70735, I70738, I70739, I70741, I70742, I70743, I70744, I70745, I70748, I70749, I7075, I70761, I70762, I70763, I70768, I70769, I70791, I70792, I70793, I70798, I70799, I708, I7090, I7091, I7092, I7100, I7101, I71010, I71011, I71012, I71019, I7102, I7103, I711, I7110, I7111, I7112, I7113, I712, I7120, I7121, I7122, I7123, I713, I7130, I7131, I7132, I7133, I714, I7140, I7141, I7142, I7143, I715, I7150, I7151, I7152, I716, I7160, I7161, I7162, I718, I719, I720, I721, I722, I723, I724, I725, I726, I728, I729, I7301, I731, I7381, I7389, I739, I7401, I7409, I7410, I7411, I7419, I742, I743, I744, I745, I748, I749, I75011, I75012, I75013, I75019, I75021, I75022, I75023, I75029, I7581, I7589, I770, I771, I772, I773, I774, I775, I776, I7770, I7771, I7772, I7773, I7774, I7775, I7776, I7777, I7779, I77810, I77811, I77812, I77819, I7782, I7789, I779, I780, I781, I788, I789, I790, I791, I798, K551, Z95820, Z95828 |
| Prior CABG | Z951 |
| Prior Myocardial Infarction | I252 |
| Prior PCI | Z955, Z9861 |
| Prior Stroke | I69, I690, I6900, I6901, I69010, I69011, I69012, I69013, I69014, I69015, I69018, I69019, I6902, I69020, I69021, I69022, I69023, I69028, I6903, I69031, I69032, I69033, I69034, I69039, I6904, I69041, I69042, I69043, I69044, I69049, I6905, I69051, I69052, I69053, I69054, I69059, I6906, I69061, I69062, I69063, I69064, I69065, I69069, I6909, I69090, I69091, I69092, I69093, I69098, I691, I6910, I6911, I69110, I69111, I69112, I69113, I69114, I69115, I69118, I69119, I6912, I69120, I69121, I69122, I69123, I69128, I6913, I69131, I69132, I69133, I69134, I69139, I6914, I69141, I69142, I69143, I69144, I69149, I6915, I69151, I69152, I69153, I69154, I69159, I6916, I69161, I69162, I69163, I69164, I69165, I69169, I6919, I69190, I69191, I69192, I69193, I69198, I692, I6920, I6921, I69210, I69211, I69212, I69213, I69214, I69215, I69218, I69219, I6922, I69220, I69221, I69222, I69223, I69228, I6923, I69231, I69232, I69233, I69234, I69239, I6924, I69241, I69242, I69243, I69244, I69249, I6925, I69251, I69252, I69253, I69254, I69259, I6926, I69261, I69262, I69263, I69264, I69265, I69269, I6929, I69290, I69291, I69292, I69293, I69298, I693, I6930, I6931, I69310, I69311, I69312, I69313, I69314, I69315, I69318, I69319, I6932, I69320, I69321, I69322, I69323, I69328, I6933, I69331, I69332, I69333, I69334, I69339, I6934, I69341, I69342, I69343, I69344, I69349, I6935, I69351, I69352, I69353, I69354, I69359, I6936, I69361, I69362, I69363, I69364, I69365, I69369, I6939, I69390, I69391, I69392, I69393, I69398, I698, I6980, I6981, I69810, I69811, I69812, I69813, I69814, I69815, I69818, I69819, I6982, I69820, I69821, I69822, I69823, I69828, I6983, I69831, I69832, I69833, I69834, I69839, I6984, I69841, I69842, I69843, I69844, I69849, I6985, I69851, I69852, I69853, I69854, I69859, I6986, I69861, I69862, I69863, I69864, I69865, I69869, I6989, I69890, I69891, I69892, I69893, I69898, I699, I6990, I6991, I69910, I69911, I69912, I69913, I69914, I69915, I69918, I69919, I6992, I69920, I69921, I69922, I69923, I69928, I6993, I69931, I69932, I69933, I69934, I69939, I6994, I69941, I69942, I69943, I69944, I69949, I6995, I69951, I69952, I69953, I69954, I69959, I6996, I69961, I69962, I69963, I69964, I69965, I69969, I6999, I69990, I69991, I69992, I69993, I69998, Z8673 |
| Right and Left Heart Catheter | 4A023N8 |
| STEMI | I2101, I2102, I2109, I2111, I2119, I2121, I2129, I213, I220, I221, I228, I229 |
| Stroke | I6300, I63011, I63012, I63013, I63019, I6302, I63031, I63032, I63033, I63039, I6309, I6310, I63111, I63112, I63113, I63119, I6312, I63131, I63132, I63133, I63139, I6319, I6320, I63211, I63212, I63213, I63219, I6322, I63231, I63232, I63233, I63239, I6329, I6330, I63311, I63312, I63313, I63319, I63321, I63322, I63323, I63329, I63331, I63332, I63333, I63339, I63341, I63342, I63343, I63349, I6339, I6340, I63411, I63412, I63413, I63419, I63421, I63422, I63423, I63429, I63431, I63432, I63433, I63439, I63441, I63442, I63443, I63449, I6349, I6350, I63511, I63512, I63513, I63519, I63521, I63522, I63523, I63529, I63531, I63532, I63533, I63539, I63541, I63542, I63543, I63549, I6359, I636, I638, I6381, I6389, I639 |
| Vascular Complications | S36899A, T81718A, T81719A, T8172XA, T81710A, T81711A, T801XXA, I770, S2500XA, S2501XA, S2502XA, S2509XA, S3500XA, S3501XA, S3502XA, S3509XA, S75011A, S75012A, S75019A, S75021A, S75022A, S75029A, S75099A, I97410, I97411, I97418, I9742, I97610, I97611, I97618, I97620, I9751, I9752, T8171, T8172, S25499A, S3559XA, S45001A, S45099A, S75001A, S75199A, S85001A, S85599A, T81710, T81710A, T8172, T8172XA |

CABG= coronary artery bypass graft, NSTEMI= non-ST segment elevation myocardial infarction, STEMI= ST segment elevation myocardial infarction, ECMO= extracorporeal membrane oxygenation, mAFP= microaxial flow pump, PCI= percutaneous coronary intervention.

**Supplementary Table 2. Baseline Characteristics of PCI Patients with ST Elevation Myocardial Infarction with and without Cardiogenic Shock**

| **Variables** | **STEMI No Shock**  **(n=127,100)** | **STEMI with Shock**  **(n=20,692)** | ***P* value** |
| --- | --- | --- | --- |
| **Age (years, mean SD)** | **64.4 (12.8)** | **67.0 (12.6)** | **<0.001** |
| **Age categories (years, %)**  **<75**  **≥75** | **106,128 (83.5%)**  **20,972 (16.5%)** | **15,568 (75.2%)**  **5,124 (24.8%)** | **<0.001** |
| **Female (%)** | **34,772 (27.4%)** | **6,538 (31.6%)** | **<0.001** |
| **Chronic Kidney Disease (%)** | **15,347 (12.1%)** | **4,931 (23.8%)** | **<0.001** |
| **Diabetes Mellitus (%)** | **50,796 (40.0%)** | **10,575 (51.1%)** | **<0.001** |
| **COPD (%)** | **10,653 (8.4%)** | **2,748 (13.3%)** | **<0.001** |
| **Peripheral Vascular Disease (%)** | **8,825 (6.9%)** | **2,896 (14.0%)** | **<0.001** |
| **Prior Stroke/TIA (%)** | **8,191 (6.4%)** | **1,893 (9.2%)** | **<0.001** |
| **Prior Myocardial Infarction (%)** | **15,101 (11.9%)** | **2,633 (12.7%)** | **<0.001** |
| **Prior PCI (%)** | **14,249 (11.2%)** | **2,528 (12.2%)** | **<0.001** |
| **Prior CABG Procedure (%)** | **3,859 (3.0%)** | **761 (3.7%)** | **<0.001** |
| **Transfer from outside hospital (%)** | **29,610 (23.3%)** | **5,331 (25.8%)** | **<0.001** |

**SD=Standard Deviation; COPD=Chronic Obstructive Pulmonary Disease; TIA=transient ischemic attack; PCI= percutaneous coronary intervention; CABG=coronary artery bypass graft; STEMI= ST-Elevation Myocardial Infarction.**

***Data are presented as mean (SD) for continuous measures, and n (%) for categorical measures.**

**Chi-square test for categorical variables/t-test for continuous variables.**

**Data from the Vizient Clinical Data Base used by permission of Vizient, Inc. All rights reserved.**

**Supplementary Table 3. Therapeutic Strategies and Outcomes for Patients with ST Elevation Myocardial Infarction who underwent PCI, complicated by cardiogenic shock**

| **Variables** | **Age < 75 years old**  **(N=15,568)** | **Age ≥ 75 years old**  **(N=5,124)** | **P value** |
| --- | --- | --- | --- |
| Right heart cath or PA catheter insertion any time during hospitalization (%) | 3,744 (24.1%) | 1,038 (20.3%) | <0.001 |
| CABG (%) | 783 (5.0%) | 141 (2.8%) | <0.001 |
| Intraaortic balloon pump (%) | 6,058 (38.9%) | 1,977 (38.6%) | 0.674 |
| mAFP (%) | 3,574 (23.0%) | 896 (17.5%) | <0.001 |
| ECMO (%) | 1,255 (8.1%) | 78 (1.5%) | <0.001 |
| Bleeding (%) | 4,618 (29.7%) | 1,430 (27.9%) | 0.017 |
| Vascular Complications (%) | 422 (2.7%) | 149 (2.9%) | 0.455 |
| Stroke (%) | 853 (5.5%) | 204 (4.0%) | <0.001 |
| Hemodialysis (%) | 1,822 (11.7%) | 461 (9.0%) | <0.001 |
| Mortality (%) | 3,894 (25.0%) | 2,055 (40.1%) | <0.001 |

CABG= coronary artery bypass graft, ECMO= extracorporeal membrane oxygenation, mAFP= microaxial flow pump, PCI= percutaneous coronary intervention.
